# A Place to Call Home: An Analysis of the Bacterial Communities in Two *Tethya rubra* Samaai and Gibbons 2005 Populations in Algoa Bay, South Africa

**DOI:** 10.3390/md15040095

**Published:** 2017-03-25

**Authors:** Samantha C. Waterworth, Meesbah Jiwaji, Jarmo-Charles J. Kalinski, Shirley Parker-Nance, Rosemary A. Dorrington

**Affiliations:** 1 Department of Biochemistry and Microbiology, Rhodes University, Grahamstown 6139, South Africa; samche42@gmail.com (S.C.W.); meesbahjiwaji@gmail.com (M.J.); j.kalinski@gmx.de (J.-C.J.K.); shirley@saeon.co.za (S.P.-N.); 2 Elwandle Coastal Node, South African Environmental Observation Network, Port Elizabeth 6001, South Africa

**Keywords:** marine microbiology, bioactive compounds, marine metagenomics, drug discovery and development

## Abstract

Sponges are important sources of bioactive secondary metabolites. These compounds are frequently synthesized by bacterial symbionts, which may be recruited from the surrounding seawater or transferred to the sponge progeny by the parent. In this study, we investigated the bacterial communities associated with the sponge *Tethya rubra* Samaai and Gibbons 2005. Sponge specimens were collected from Evans Peak and RIY Banks reefs in Algoa Bay, South Africa and taxonomically identified by spicule analysis and molecular barcoding. Crude chemical extracts generated from individual sponges were profiled by ultraviolet high performance liquid chromatography (UV-HPLC) and subjected to bioactivity assays in mammalian cells. Next-generation sequencing analysis of 16S rRNA gene sequences was used to characterize sponge-associated bacterial communities. *T. rubra* sponges collected from the two locations were morphologically and genetically indistinguishable. *C*hemical extracts from sponges collected at RIY banks showed mild inhibition of the metabolic activity of mammalian cells and their UV-HPLC profiles were distinct from those of sponges collected at Evans Peak. Similarly, the bacterial communities associated with sponges from the two locations were distinct with evidence of vertical transmission of symbionts from the sponge parent to its embryos. We conclude that these distinct bacterial communities may be responsible for the differences observed in the chemical profiles of the two Algoa Bay *T. rubra* Samaai and Gibbons 2005 populations.

## 1. Introduction

Sponges (phylum: Porifera) are ancient extant metazoan species that prey on microbes in surrounding seawater by filtering large volumes of seawater [1,2]. This puts the sponge into direct contact with a diverse community of microorganisms. Some of these bacteria evade predation and are, instead, recruited by the sponge as symbionts (horizontal transfer). Alternatively, bacterial sponge symbionts are transferred to sponge progeny from the parent sponge (vertical transfer) and are, therefore, present in the nascent sponges [3]. Sponge bacterial symbionts promote sponge fitness by playing a role in carbon, nitrogen, and sulfur nutrient cycling [4,5,6]. The symbiotic bacteria may also be the source of bioactive secondary metabolites that provide the sponge with a chemical defense against predation, fouling, and diseases [6,7,8,9,10].

The genus *Tethya* Lamarck 1815 is the largest in the Tethyidae family, including 92 shallow, warm and temperate to cold and deep water species [11,12]. Records indicate that *Tethya* species also inhabit caves [13], the Arctic Ocean [14], and have been collected from depths of 805 m [12]. *Tethya* sponges are hemispehical or spherical with a distinct cortex region surrounding the inner choanosomal region which consists of large radial megascleres [12].

*Tethya* species are known to produce bioactive secondary metabolites [15,16]. Two nucleosides, spongothymidine and spongosine, were isolated from the sister genus, *Tectitethya crypta* [17,18]. The synthetic analogue, Cytarabine is used as a chemotherapy drug in the treatment of leukemia and non-Hodgkin’s Lymphoma [19], while another, Vidarabine, is an antiviral compound [20]. Recently, the biosynthetic origin of spongosine and other structurally-related compounds was found to be a strain of *Vibrio harveyi* that was associated with *T. crypta* [21]. Bioactive compounds have also been isolated from fungi associated with *Tethya* species [22,23]. In this study we investigated the bacterial communities associated with *Tethya rubra* Samaai and Gibbons 2005 [24], which is common at depths between 30 and 51 m on reefs in Algoa Bay, South Africa. In addition, we compared the chemical profiles of crude extracts prepared from the *Tethya rubra* Samaai and Gibbons 2005 sponges. In this article, we report that *T. rubra* sponges collected from different locations in Algoa Bay have distinct sponge-associated bacterial communities that are transferred from parent to progeny embryos. In addition, we report that the chemical profiles of crude extracts prepared from the *Tethya rubra* Samaai and Gibbons 2005 sponges collected from different locations were distinct.

## 2. Results

### 2.1. Identification of Sponge Specimens

**Figure 1:**
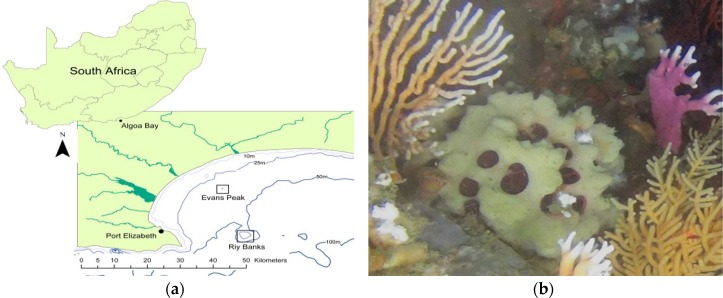

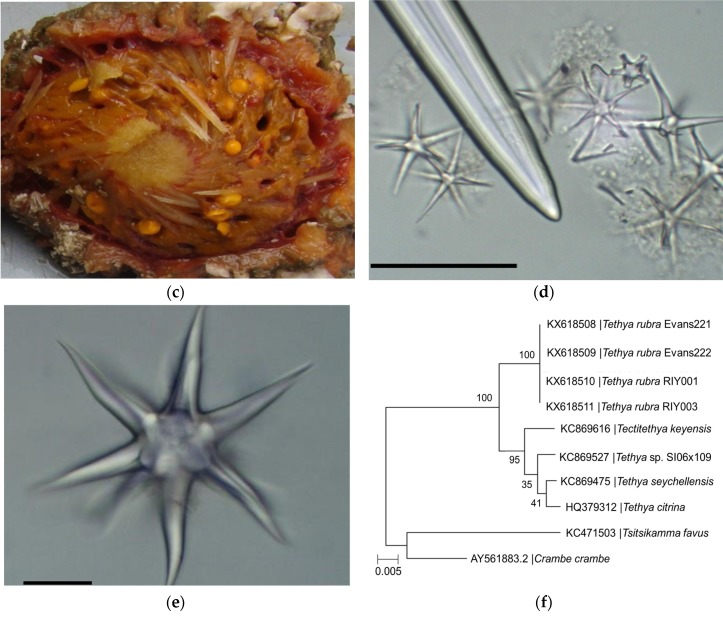
Morphological and molecular identification of *Tethya rubra* Samaai and Gibbons 2005 specimens collected from Algoa Bay. (**a**) Geographic location of sponge collection sites; (**b**) in situ image of a live specimen collected from RIY Banks with an encrusting *Mycale* sp. and white softcoral over the surface of the *T. rubra* sponge; (**c**) *T. rubra* specimen with bright yellow embryos visible in the endosome and surrounded by a well-defined cortex that is encrusted with epibionts; (**d**) *T. rubra* skeletal components consisting of the distal end of an anisostrongyloxea megasclere, several small oxyspheraster, and single micraster microxyspheraster; (**e**) large oxyspheraster. Scale bars represent 50 µm; and (**f**) phylogenetic analyses of *T. rubra* sponge specimens based on a partial sequence of the 28S rRNA gene sequences aligned with Clustal W and constructed using the maximum likelihood method with 500 bootstrap replicates in MEGA7 [26]. Scale bar represents an estimated 0.5% sequence substitution. GenBank accession numbers are included in the image.

Two sponge specimens (Evans221 and Evans222) were collected by a remote operating vehicle (ROV) from the Evans Peak reef in November 2015 and two specimens (RIY001 and RIY003) were collected by ROV from the RIY Banks reef in February 2016. Both are sites are in Algoa Bay, Port Elizabeth, South Africa (Figure 1a,b). Evans Peak is located inshore, in shallow water, and influenced by the inflow of nutrient-rich freshwater from two nearby estuaries. In contrast, RIY Banks reef is situated offshore at the edge of the coastal shelf, and is impacted by upwelling of cold bottom water and incursions by the Agulhas current (Figure 1a). All four sponge specimens were morphologically identified as *Tethya rubra* Samaai and Gibbons 2005 through examination of gross morphology and spicule analyses (Figure 1c–e). Dissection of the sponges revealed a number of bright yellow, round bodies in the endosome [25] (or choanosome [24]) of the sponges that were identified as embryos (Figure 1c). A 600 nt DNA fragment of the 28S rRNA gene (GenBank asscession numbers KX618508–KX618511) was amplified by PCR from each sponge as well as embryos isolated from the sponges, analysed by Sanger sequencing, and used for taxonomic identification (data not shown) and phylogenetic analyses of the sponges (Figure 1f).

### 2.2. Biological Activity Assays

**Figure 2:**
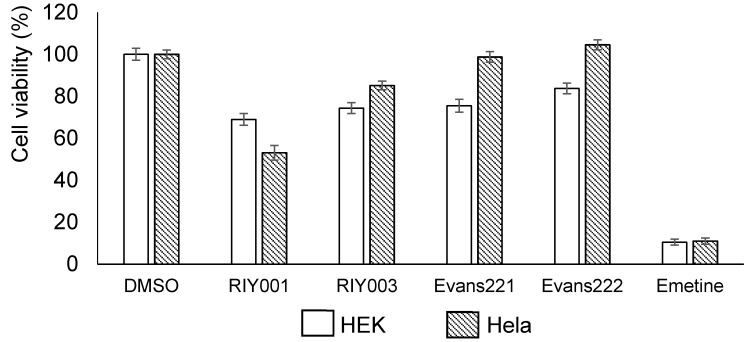
Bioactivity of *T. rubra* chemical extracts. HEK293 and HeLa cells (1 × 10^6^ cells) were exposed to crude extracts from the *T. rubra* sponges for a 48 h period before bioactivity was quantified. The crude extracts were used at a final concentration of 20 µg per mL. The carrier, DMSO (treatment volume 1 µL), was included as a negative control, and normalized to represent 100% cell viability. Emetine (Sigma E2375-1g), a cytotoxic alkaloid, was used at a final concentration of 25 ng per mL as a positive control in these assays.

Chemical extracts were generated from frozen sponge material by extraction in a mixture of dichloromethane (DCM) and methanol (MeOH) overnight. The extracts were dried in vacuo and then resuspended in DMSO for bioactivity assays or MeOH for ultraviolet high performance chromatography (UV-HPLC) profiling. The bioactivity assays involve the use of a blue resazurin-based compound that is reduced by viable cells to a pink-colored compound, while there is a concomitant increase in the levels of a fluorescent intermediate by viable cells. These reactions can be used to indicate the cytotoxicity of the compounds of interest on the mammalian cells. Human embryonic kidney (HEK) 293 and human cervical cancer (HeLa) cells were treated with crude extracts of compounds isolated from *T. rubra* sponges at a final concentration of 20 µg·mL^−1^. Mild cytotoxicity was observed overall, with the crude extract from RIY001 exhibiting the greatest levels of cytoxicity (Figure 2). Crude extracts from all four sponges exhibited mild cytotoxicty against HEK293 cells whilst only extracts from RIY001 and RIY003 exhibited significant cytotoxic effects on Hela cells.

**Figure 3:**
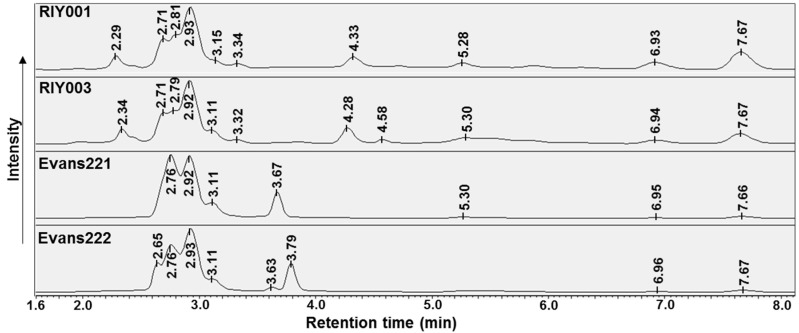
UV-HPLC (250 nm) chromatograms of chemicals isolated by DCM:MeOH extracts prepared from *T. rubra* sponge material. The mobile phase consisted of MeOH:H_2_O (1:1) at pH 3.0 and retention times are annotated in minutes.

The chemical profiles of crude extracts isolated from the *T. rubra* sponges, analyzed by UV-HPLC, indicated strong similarity between the peak profiles of samples from the same location. A common set of peaks appear to be produced by all extracts (Figure 3); these peaks are evident at retention times between 2.6 min, 3.2 min and above 5 min, albeit lower intensities were observed for the late eluting peaks in extracts from Evans Peak. Crude extracts from sponges collected at different locations, namely RIY Banks versus Evans Peak, showed distinct inter-location differences between the sponges. Unique to specimens RIY001 and RIY003 were peaks eluting at approximately 2.3 min, 3.3 min, and 4.3 min. Likewise, extracts of specimens from Evans Peak showed peaks between 3.6 min and 3.8 min that were not observed in extracts generated from specimens collected at RIY Banks. These results indicate that there may be two populations of *T. rubra* sponges that exhibit distinct chemical profiles dependent on their geographic location.

### 2.3. Microbial Community Analysis

Total DNA isolated from sponge material was subjected to PCR using primers specific for the V4–V5 region of the bacterial 16S rRNA gene sequence. The amplified DNA was analyzed by pyrosequencing. A total of 151,002 sequence reads of approximately 450 nt in length were used for a comparative analysis of the bacterial communities that were present in the cortex, endosome, and embryos of the *T. rubra* sponges, as well as those present in the surrounding water column. Rarefaction analysis indicated that the sponge bacteria were sampled almost to completion at the species level (data not shown). Species richness of samples was calculated using the abundance-based coverage estimator (ACE) method and was found to be lower in both sponge and water samples collected from RIY Banks relative to samples collected from Evans Peak.

**Figure 4:**
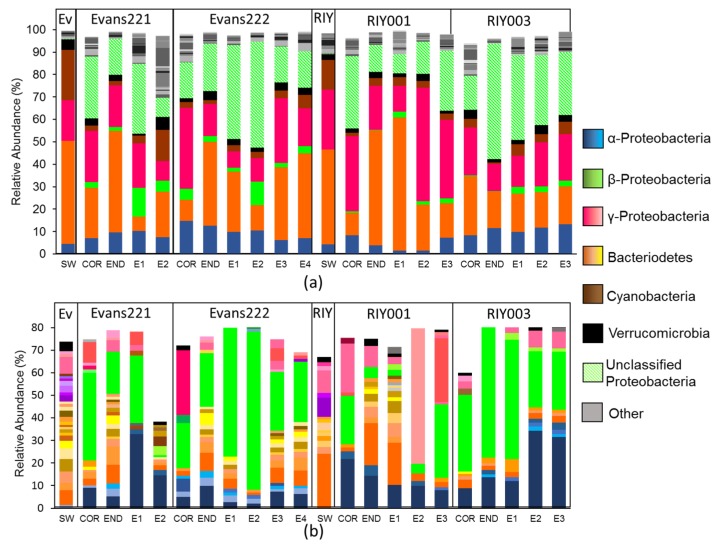
Characterization of the bacterial communities present in *T. rubra* sponges collected from Algoa Bay. (**a**) Phylogenetic classification of amplicon reads using the SILVA bacterial database (version 123) as reference (**b**) bacterial OTUs clustered at a distance of 0.03 with a relative abundance greater than 1%. Read abundance is indicated as the relative percentage of the total bacterial reads analyzed for each sponge. OTUs within a phylum/class were identified with BLAST using the GenBank database. The phylogenetic classification of individual OTUs is indicated in varying shades of the same color. SW: Seawater collected from Evans Peak (Ev) or RIY Banks (RIY). COR: Cortex, END: Endosome, E: Embryo.

Phylogenetic classification of amplicon libraries showed that at the phylum level, the bacterial populations in the cortex, endosome and embryos of the *T. rubra* sponges were similar to one another with dominant populations of Bacteriodetes and Gammaproteobacteria (Figure 4a). This was also the case for the water column. However, *T. rubra* sponges harbored a large number of unclassified Proteobacteria, as well as some Betaproteobacteria that were poorly represented in the surrounding seawater (Figure 4a).

Sequence reads clustered into operational taxonomic units (OTUs) at a distance of 0.03 revealed a similar pattern to that which was observed in the phylogenetic classification (Figure 4b), and a single Betaproteobacterial OTU, identified using BLAST against the GenBank database, appeared to be dominant. Further inspection revealed that this OTU represented the majority of the reads classified by the SILVA database as “Unclassified proteobacteria”. The Betaproteobacterium OTU, as well as an Alphaproteobacterium OTU, were present in all sponge tissues and the embryos, but not in the surrounding seawater from either of the collection sites.

**Figure 5:**
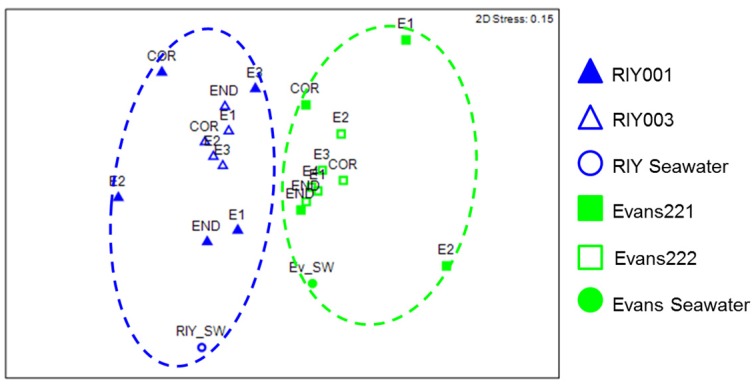
Statistical oordination of unique (0.00) bacterial communities in *T. rubra* sponges using non-metric multidimensional scaling between the collection sites; RIY Banks (blue) and Evans Peak (green). Sponge/embryo samples from RIY Banks are represented by triangles and sponge/embryo samples from RIY Banks are represented by squares. Water samples are represented with circles. Square root transformation was applied to bacterial community abundance data prior to construction of Bray-Curtis resemblance matrix based on partial 16S rRNA gene sequence. (2D stress value: 0.14). Green and blue dotted circles represent statistically significant clusterings (*p* < 0.05) determined using the ANOSIM option in PRIMER7 (Quest Research Limited, Auckland, New Zealand). SW: Seawater collected from Evan’s Peak (Ev) or RIY Banks (RIY). COR: Cortex, END: Endosome, E: Embryo.

We analyzed the relative abundance of unique OTUs (at a distance of 0.00) to more closely examine the bacterial communities in each of the sponges. A nonmetric multidimensional scaling (NMDS) plot ordination of pairwise distances was generated, followed by an ANOSIM analysis to compare the bacterial communities relative to a collection site (Figure 5). The diversity of sponge-associated bacterial communities and those in the water differed significantly between the two sites (*p* < 0.05) and the relationship between bacterial community composition and collection site was moderate (*r* = 0.473).

**Figure 6:**
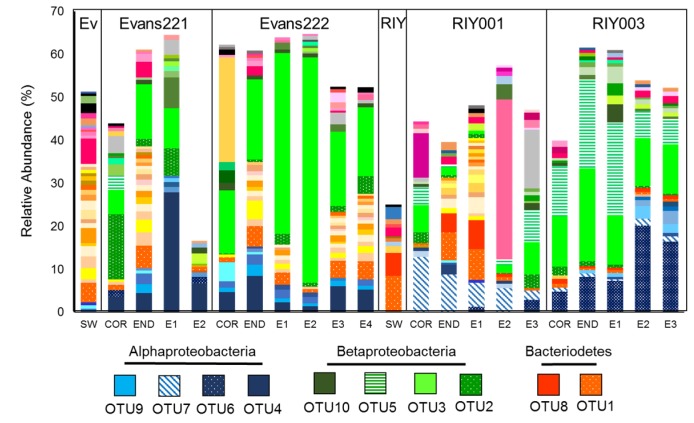
Dominant bacterial OTUs (0.00) identified in *T. rubra* sponges and seawater collected from Evans Peak and RIY Banks. Unique OTU read abundance was measured relative to the total number of reads present in each sample and only OTUs with a relative abundance greater than 0.5% were plotted. OTUs were colored per phyla/class as in previous figures. SW: Seawater, COR: Cortex, END: Endosome, E: Embryo, Ev: Evans Peak, RIY: RIY Banks.

Further analysis of the unique OTUs revealed that a single Bacteriodetes strain, OTU1, was ubiquitous in all the sponges, their embryos and in the surrounding seawater at both sites (Figure 6). Two Betaproteobacterium strains; OTU2 and OTU3, were sponge-specific, being present in all the sponges and their embryo samples, but not in the water column, regardless of the collection site. A third betaproteobacterial strain, OTU5, was dominant in the RIY Banks sponges compared with those from Evans Peak. Most striking were the differences observed in the distribution of sponge-associated Alphaproteobacteria. OTU4 was present in both sponge and embryonic tissue from samples collected at Evans Peak, but absent in the surrounding seawater. Similarly, OTU6 and OTU7 were dominant in RIY003 and RIY001 tissues, respectively, present in low abundance, or absent, in the Evans Peak sponges, and absent from the surrounding water column. The presence of these betaproteobacterial and alphaproteobacterial OTUs in the embryos of sponges from the same site lead us to conclude that they are inherited by vertical transmission from the parent sponge. Other symbionts, e.g., OTU1, are recruited from the surrounding seawater.

**Figure 7:**
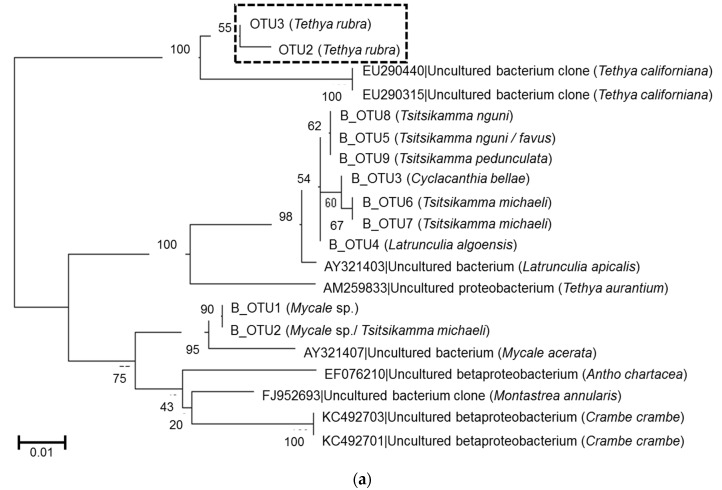

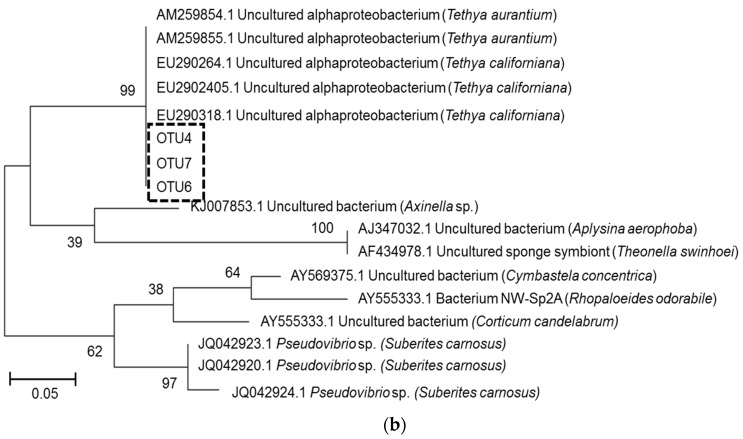
Phylogeny of *T. rubra*-associated bacterial OTUs (0.00). (**a**) Conserved Betaproteobacteria OTUs and (**b**) conserved Alphaproteobacterial OTUs based on partial 16S rRNA gene sequences (of the V4–V5 region) aligned with Clustal W and inferred using the maximum likelihood method with 500 bootstrap replicates in MEGA7. Conserved OTUs identified in this study are highlighted by the boxes. Scale bar represents an estimated 1% and 5% sequence substitution respectively.

Previous studies on latrunculid sponges collected from the same locations showed the presence of conserved Betaproteobacterium species [27]. A phylogenetic comparison between the *T. rubra* and latrunculid betaproteobacterial OTUs showed that the *T. rubra* OTUs were most closely related to Betaproteobacteria isolated from *Tethya californiana* and only distantly related to the betaproteobacterial OTUs associated with latrunculid sponges collected from RIY Banks and Evans Peak (Figure 7a). Similarly, the *T. rubra* alphaproteobacterial OTUs clustered in a *Tethya*-specific clade including OTUs from *T. aurantium* and *T. californiana* (Figure 7b).

## 3. Discussion

In this study, we show that *T. rubra* sponges in Algoa Bay have conserved bacterial consortia, including dominant alpha- and betaproteobacterial species that were not detected in the surrounding seawater. Closer inspection showed that different strains of these conserved symbionts are dominant in the sponges collected from RIY Banks versus those that were collected from Evans Peak. The presence of the same microbial consortia in the embryos suggests that there is active vertical transmission of these alpha- and betaproteobacterial symbionts from the parent sponges to their progeny and that these bacteria may play a vital role in the survival of the *T. rubra* sponges in the competitive marine environments.

The differences observed between the microbial communities in sponges collected from Evans Peak and RIY Banks may be due to seasonal variations in the water column and/or the environmental factors relating to the geographic location of the two reefs. In a previous study we found no evidence of seasonal variation in the bacterial communities associated with *Tsitsikamma favus* sponges collected from Evans Peak [27]. Furthermore, a statistical analysis of the bacterial communities from four seawater samples collected at Evans Peak between August 2014 and June 2016 showed that there was no significant difference (*p* = 0.71) between them (S.H. Hilliar and R.A. Dorrington, unpublished data). Therefore, while seasonal differences cannot be ruled out, we believe that the differences observed between associated microbiota of the Evans Peak and RIY Banks *T. rubra* sponges are more likely to be due to their geographical location. While we cannot fully account for why these communities are different, an important observation is that they are associated with distinct chemical profiles in the two sponge populations.

Symbiotic Betaproteobacteria that have co-evolved with their hosts are known to associate with many sponge species from diverse families including the Latrunculiidae, Mycalidae, Crambeidae, and Tethyidae [27,28,29,30,31]. Amongst the *Tethya* species, *T. californiana*, *T. aurantium*, and *T. stolonifera* have all been shown to harbor sponge-specific, temporally-stable betaproteobacterial species that are represented by a single, dominant OTU or DGGE band [32,33,34]. The *T. rubra* betaproteobacterial OTUs are most closely related to sponge-specific Betaproteobacteria of *T. californiana* and only distantly related to that of *T. aurantium* suggesting that *T. rubra* may be phylogenetically related to *T. californiana*. Interestingly, the *T. rubra* OTUs were also only distantly related to Betaproteobacteria identified in Latrunculid sponges that were collected from RIY Banks and Evans Peak supporting the hypothesis that these symbionts are vertically transmitted from parent to progeny sponges. A recent study investigating the genome of a betaproteobacterium from *Amphimedon queenslandica* suggests that this symbiont forms part of a dual-symbiotic relationship with a sulfur-oxidizing Gammaproteobacterium and the authors present evidence for possible pathway complementation and resource portioning in the sponge [31].

The *T. rubra*-associated microbiome also showed the presence of an Alphaproteobacterium species. As with the Betaproteobacterium, this species is also phylogenetically related to Alphaproteobacteria from *T. aurantium* and *T. californiana*, suggesting a common lineage and a conserved symbiotic interaction with the *Tethya* host. The *T. rubra* Alphaproteobacterium includes three strains; the distribution and relative abundances of which were different in sponges from RIY Banks (OTU6 and OUT7) compared to Evans Peak (OTU4). It is interesting to note that this species of Alphaproteobacteria is not present in sympatric Latrunculid sponge species collected from the same reef. Vertically transferred sponge-associated Alphaproteobacteria have been implicated in the production of bioactive compounds [34,35,36]. For these reasons, it is tempting to speculate that these Alphaproteobacteria may play a role in the biosynthesis of the compounds that contribute to the distinct chemical profiles that we observed in sponges collected from the two locations of Algoa Bay. We are currently engaged in the characterization of these chemical compounds and their biological targets.

## 4. Materials and Methods

### 4.1. Sample Collection

Sponge specimens were collected using a SAAB Seaeye Falcon 12177 remote operated vehicle (ROV) (SAAB Seaeye, South Hampton, UK) in Algoa Bay, Port Elizabeth, South Africa. Specimens TIC2015-221 (Evans221) and TIC2015-222 (Evans222) were collected from Evans Peak (33°50.729 S, 25°48.998 E) at a depth of approximately 30 m. Specimens TIC2016-001 (RIY001) and TIC2016-003 (RIY003) were collected at RIY Banks (34°00.099 S, 25°51.373 E) at a depth of 51 m. A volume of 5 L of water was also collected from each site and filtered using 0.22 μm water filters (MoBio, Cat. No. 14880-50-WF). All samples were stored in RNALater^®^ (ThermoFisher Scientific, Johannesburg, South Africa) at −80 °C.

### 4.2. Sample Processing and DNA Extraction

Sponge samples were washed in artificial seawater (ASW) (24.6 g NaCl, 0.67 g KCl, 1.36 g CaCl_2_·2H_2_O, 6.29 g MgSO_4_·7H_2_O, 4.66 g MgCl_2_·6H_2_O, and 0.18 g NaHCO_3_ per 1 L) and all other visible organisms inhabiting the sponge were aseptically removed using forceps. Wedges approximately 1 cm^3^ were aseptically cut from each sponge sample and the endosome and cortex tissue separated by hand. Embryos were removed and individually washed in ASW for 5 min using a vortex set at a low speed. Each tissue type was macerated using a mortar and pestle in 1 mL ASW and then clarified by centrifugation at 13,000 rpm for 2 min; the supernatant was removed. Genomic DNA was extracted from the sponge pellets using the ZR Soil Microbe DNA MiniPrep kit (Zymo Research, Cat. No. D6001) (Irvine, CA, USA). DNA was also extracted from one-half of a water filter using the PowerWater DNA Isolation kit (Cat. No. 14900-S) (MoBio, San Diego, CA, USA).

### 4.3. Identification of Sponge Specimens

Sponge taxonomy was determined by analysis of the gross morphology and the spicules. Identification was confirmed by Sanger sequencing of the D3-D5 region of the 28S rRNA gene fragment using primer pair RD3a (5’-GAC CCG TCT TGA AAC ACG A-3’) and RD5b2 (5’-ACA CAC TCC TTA GCG GA-3’) [37]. Each PCR amplification reaction included 0.3 μM of each primer, 0.75 mM dNTPs, 1X Buffer with MgCl_2_, 0.5 U Kapa HiFi Taq Polymerase, and 50 ng of template DNA in a total volume of 25 μL. Cycling parameters were as follows: initial denaturation at 95 °C for 5 min, followed by 35 cycles of 94 °C for 30 s, 45 °C for 20 s, 72 °C for 1 min, with a final extension of 72 °C for 10 min. Phylogenetic trees were constructed using the MEGA7 software [26] using ClustalW and Maximum-likelihood methods, with 500 bootstrap replicates. Comparative sponge 28S rRNA gene sequences were obtained from the NCBI nucleotide database (http://www.ncbi.nlm.nih.gov/).

### 4.4. Generation of Crude Extracts

Approximately 4 g (dry weight after extraction) of frozen sponge material was soaked in 150 mL DCM:MeOH (2:1) overnight. The extracts were dried in vacuo. These dried crude extracts were used for both bioactivity assays as well as UV-HPLC analysis.

### 4.5. Bioactivity Assays

Human embryonic kidney (HEK293) and human cervical cancer (HeLa) cells were maintained in DMEM supplemented with 4 mM L-glutamine and 10% FBS at 37 °C in an atmosphere that contained 5% CO_2_. HEK293 and HeLa cells were plated at a density of 1 × 10^6^ cells per 1 mL in each well of a 24 well cell culture plate (NEST, Cat. No. 702001) and settled overnight. The freeze dried crude extracts were resuspended in DMSO to a concentration of 20 mg per mL. A total of 1 µL of this solution was used per mL of assay volume resulting in a final crude extract concentration of 20 µg per mL in each assay. The resazurin-based in vitro toxicology assay kit (Cat. No. R6892) (Sigma Aldrich, Saint Louis, MO, USA) was used to evaluate the bioactivity, and by inference, the cytotoxicity, of the bioactive compounds in the crude extracts after 48 h.

### 4.6. UV-HPLC Analysis

The crude extracts from the *T*. *rubra* sponges were re-suspended at 1 mg per mL in MeOH for HPLC analysis, which was facilitated at 1.1 mL per min with the mobile phase MeOH:H_2_O (1:1) +0.05% formic acid, an injection volume of 10 µL and a detector wavelength of 250 nm. Analysis was performed on a Waters HPLC system with a Waters 1525 binary pump, 4.6 × 250 mm Xbridge BEH RP18 column and a Waters 2489 UV-VIS detector. All solvents used for the UV-HPLC analysis were HPLC-grade.

### 4.7. Amplicon Library Preparation and Pyrosequencing

Amplicon libraries of 16S rRNA gene fragments (V4-V5) were generated using MID-tagged primer pair E517F (5’-CAG CAG CCG CGG TAA-3’) and E969-984 (5’-GTA AGG TTC YTC GCG T-3’) [38,39]. PCR reactions consisted of 0.3 μM of each primer, 0.75 mM dNTPs, 1X Buffer with MgCl_2_ and 0.5 U Kapa HiFi Taq Polymerase in a 50 μL reaction. Cycling parameters were as follows: initial denaturation at 98 °C for 5 min, five cycles of 98 °C for 45 s, 45 °C for 30 s, 72 °C for 45 s, followed by 18 cycles of 98 °C for 30 s, 50 °C for 30 s, 72 °C for 45 s, with a final extension of 72 °C for 5 min. Amplified DNA was gel purified using the Isolate II PCR and Gel Kit (Cat. No. BIO-52060) (Bioline, London, UK). Amplicons were subjected to a second purification using AMPure XP beads (Agencourt, Beckman-CoulterLife Science, Johannesburg, South Africa) before the resultant amplicon libraries were sequenced with the GS Junior Titanium Sequencing platform (454 Life Sciences, Roche, Branford, CT, USA) as per the manufacturer’s specifications (raw datasets have been deposited in the NCBI Sequence Reads Archive (SRA) database (SRP099126).

### 4.8. Sequence Data Curation and Analysis

Amplicon library sequence datasets were curated using Mothur software (Department of Microbiology & Immunology at The University of Michigan, Ann Arbor, MI, USA) [40]. Reads that were shorter than 200 bp or that included ambiguous nucleotides or homopolymeric runs longer than 7 bp, were removed from the respective datasets. Chimeric reads within the datasets were identified using the UCHIME algorithm (Version 4.2) [41] and removed. Reads were compared against the SILVA Bacterial Database (Release version 123) and those classified as unknown, chloroplast, mitochondrial, or eukaryotic DNA were removed. A distance matrix (cut-off at 0.15) was used to cluster reads into OTUs at distance values of 0.03 and 0.00. Data was converted to percentage of reads per OTU relative to the total number of reads per sample. All OTUs with a relative percentage greater than 0.5% were plotted as dominant bacterial populations. Phylogenetic trees of bacterial OTUs (0.00) were constructed in MEGA7 software [26] using ClustalW alignment and phylogeny inferred using the maximum likelihood method with 500 bootstrap replicates. Comparative bacterial 16S rRNA gene sequences were obtained from the NCBI nucleotide database (http://www.ncbi.nlm.nih.gov/) and from previous studies [27]. PRIMER7 (Quest Research Limited, Auckland, New Zealand) was used to generate a non-metric multidimensional scale (NMDS) plot using count table abundance data (generated using Mothur). This data then underwent a square root transformation prior to Bray-Curtis resemblance and ANOSIM analysis. Rarefaction analysis and measurement of richness and diversity of unique OTUs was performed with R [42] using the *vegan* package [43].

## 5. Conclusions

*T. rubra* Samaai and Gibbons 2005 sponges collected from Evans Peak and Riy Banks reefs in Algoa Bay, South Africa are associated with distinct bacterial communities with evidence of vertical transmission of symbionts from the sponge parent to its embryos. UV-HPLC profiles of chemical extracts of sponges from the two locations suggest two different chemotypes. We propose that the distinct bacterial communities may be responsible for the differences observed in the chemical profiles of the two Algoa Bay *T. rubra* Samaai and Gibbons 2005 populations.
